# Perception, pattern of use, partner support and determinants of uptake of family planning methods among women in rural communities in Southeast Nigeria

**DOI:** 10.1186/s40834-020-00120-x

**Published:** 2020-09-01

**Authors:** Ifeyinwa Chizoba Akamike, Ugochukwu Chinyem Madubueze, Ijeoma Nkem Okedo-Alex, Chika Julius Anyigor, Benedict Ndubueze Azuogu, Chukwuma David Umeokonkwo, Chinyere Ojiugo Mbachu

**Affiliations:** 1 Department of Community Medicine, Alex Ekwueme Federal University Teaching Hospital, Abakaliki, Ebonyi State Nigeria; 2 Department of Community Medicine, Ebonyi State University, Abakaliki, Nigeria; 3 Department of Community Medicine, College of Medicine, University of Nigeria, Nsukka, Nigeria

**Keywords:** Perception, Pattern of use, Partner support, Determinants, Family planning

## Abstract

**Background:**

Family planning is a cost-effective strategy for achieving population development. Family planning uptake is low in sub-Saharan Africa, including Nigeria. We assessed the perception, pattern of use, partner support and determinants of uptake of family planning methods among married women of reproductive age in rural communities of Ebonyi state.

**Methods:**

This is part of a baseline report of a quasi-experimental study. A total of 484 married women of reproductive age were recruited using multistage sampling method. Four focus group discussions (men and women) and pre-tested semi-structured interviewer-administered questionnaires were used to collect information from the participants. Data were analysed using Statistical Package for Social Sciences (SPSS) version 20 software and thematic analysis. Chi-square test and logistic regression were carried out at 5% significance level.

**Results:**

Only 26.2% of respondents were currently using any method of family planning. The most commonly used method was the natural method (57%). Amongst those who reported using artificial methods, 32.7% used condoms, 27.3% used implant while 23.64 and 16.4% used injectables and pills respectively. Predictors of current use of any family planning method were: older age (AOR = 1.7, 95%CI = 1.01–3.00), having more than five children (AOR = 1.7, 95%CI = 1.05–2.83), minimum of secondary level of education for respondent (AOR = 3.3, CI = 1.60–6.96) and their husband/partner (AOR = 2.0, 95%CI = 1.05–3.92). Qualitative findings showed that only few families were using a method of family planning and those who did not practice family planning perceived it to interfere with God’s plan for fruitfulness and to be counter-productive to household income due to decreased manpower for agricultural activities. Poor partner involvement and support for family planning was also cited as a deterrent by both male and female participants.

**Conclusions:**

Perception and use of family planning methods is poor in rural communities of Ebonyi state. Improving uptake of family planning methods in these rural communities will require proper demographic targeting as well as debunking fatalistic views, and cultural and religious myths around family planning.

## Background

Family planning allows people to attain their desired number of children and determine the spacing of pregnancies. This is achieved through the use of family planning methods and treatment of infertility [1, 2]. Family planning is voluntary, and available methods of contraception (previously referred to as birth control) can be customized to individual needs with a range of methods that are acceptable to all and effective if used correctly [2]. The choice and use of a particular family planning method and their sources vary globally. Family planning can be categorized into two: natural and artificial methods. The natural methods include abstinence, coitus interruptus, safe period, and lactational amenorrhoea. Common artificial methods used are; condoms, injectables, pills and Intra Uterine Contraceptive Devices (IUCD) [1]. Over200 million women of reproductive age in developing countries who want to avoid pregnancy are not using a modern contraceptive method [1–3]. This is due to limited choice of methods; limited access to contraception, particularly among young people, poorer segments of populations, or unmarried people; fear or experience of side-effects; cultural or religious opposition; poor quality of available services; user and provider bias and, gender-based barriers [1].

Globally, among the 1.9 billion women of reproductive age (15–49 years) living in the world in 2019, 1.1 billion have a need for family planning. Of the number who need family planning, 842 million (44%) use artificial methods of contraception, 80 million use traditional methods of family planning, while 190 million (10%) women have an unmet need for contraception [4]. In 2018, the prevalence rate for any contraceptive use among Nigerian women was 17% and this was more among sexually active single women (28%) than currently married women (12%) [5]. Although currently married women may have more sexual exposure, overall contraceptive use has only marginally increased from 15% in 2013 to 17% in 2018 while use of any modern method of contraception also increased, from 10 to 12%. In addition, there has been a slight rise in the use of implants since 2008, from 0 to 3% [5].

Some studies have reported variations in preference and pattern of use of family planning methods. In south-eastern Asia, the most common contraceptive method used was Intra-uterine device, 18.6% of women rely on this method [4]. In Europe and Northern America, the pill and female condom are the most commonly used methods (17.8 and 14.6% of women, respectively), while in Latin America and the Caribbean it is female sterilization and the pill (16.0 and 14.9%, respectively) [4]. Sub-Saharan Africa is the only region in which injectables are the dominant method with a prevalence of 9.6% among women of reproductive age [4]. A three year review of pattern of contraceptive use among women aged 15 to 52 years in southwest Nigeria revealed that majority (46.3%) preferred Jadelle implant while the least used implant was Norplant (0.5%) [6]. In that study married women were more likely to seek family planning methods and the motivating factor was based on the increasing challenge in raising children and the need to reduce family size [6].

Although there has been slight improvement in contraceptive use in Nigeria, the uptake is still very low. The Nigerian demographic health survey of 2018 reported a contraceptive uptake ranging from 2 to 7% for Ebonyi State with lower values in rural areas compared to urban areas [5]. Therefore, this study seeks to understand the perception, pattern of use, partner support and determinants of uptake of family planning methods among married women in rural areas of Ebonyi state, Nigeria. Findings from this study will be useful in informing the design of family planning interventions for married women in similar rural contexts.

## Materials and methods

### Study area

The study was conducted in Igbeagu in Izzi local government area (LGA) and Ezzamgbo in Ohaukwu LGA, both in Ebonyi State in South Eastern Nigeria. The State has a land mass of about 5935 km^2^. Majority of the people of Ebonyi State live in the rural area and are predominantly of Roman Catholic faith. About 75% of the population is involved in some form of agriculture, and this constitutes the key occupation of people in the study communities where there is a preference for crops such as rice, yam, cassava, maize and some other economic crops. The staple foods include locally prepared cassava, rice, and yam. Family planning services are provided at all levels of healthcare in the State.

### Study design

This is part of a baseline report of a quasi-experimental study. Detailed description of the study design has been published elsewhere [7].

### Study population

The study population included women of reproductive age (15–49 years) who were either married or in a union. Pregnant women were excluded from the study. Women within the reproductive age, who had achieved menopause, were also excluded.

### Sample size and sampling technique

A total of 484 women of reproductive age were approached to participate in this survey. Respondents were selected from the two rural LGAs in Ebonyi north senatorial zone using a multistage sampling technique. Detailed description of the sample size determination and sampling technique have been presented in another paper [7].

### Data collection

A pre-tested, semi structured interviewer administered questionnaire was used to collect information from respondents. Information collected include socio-demographic characteristics of the respondents such as age, level of education and spouse’s level of education; use of family planning; pattern of use including method used, frequency of use and duration of use. The focus group discussion (FGD) was used to better understand community perceptions and practices (including sources) of family planning and to explore in-depth the factors that influence acceptability and use of modern methods of family planning. A total of four FGDs were conducted, two for the men and two for the women. Participants for the FGD were married and within the reproductive age group. Men and women who are outspoken and willing to participate were identified and purposively selected with the help of community health workers. Outspoken people were selected for the FGD because of the need to explore in-depth and so required people who can articulate and voice out their opinions so as to better understand the phenomenon under study. A total of seven questions with probes were discussed. There were about 10–12 discussants per FGD. Permission was obtained from the discussants to audio record the sessions to assist the researcher obtain details of the discussion. The researcher was the moderator and a research assistant was the note-taker who recorded key issues raised in the sessions. Each FGD lasted about 35 min.

### Measurement of variables

The independent variables were: socio-demographic and household characteristics of women such as age, level of education, religion, husband’s education, number of living children, family type, household head and main decision maker on health issues. These variables were measured using the structured questionnaire. Age was measured using age as at last birthday. Mean and standard deviation were calculated for age. All the other independent variables were measured either on the nominal or ordinal scale and were later recoded into two categories. Frequencies and proportions were calculated for categorical variables. Religion was coded into two categories: Roman Catholic denomination and other denominations since only Christians participated in the survey.

The dependent variables include: pattern of use and uptake of family planning. Pattern of use was measured using self-reports of commonly used method of family planning (that is type of method used); frequency of use; and duration of use.

Uptake of modern methods of family planning refers to the use of modern methods of family planning. This was measured by: proportion currently using a method. Questions with “Yes” or “No” was used to determine respondents’ uptake of family planning.

### Data analysis

Quantitative data analysis was carried out using the Statistical Package for Social Sciences (SPSS) for the Microsoft Window version 20 software. Frequencies and proportions were calculated for categorical variables while means and standard deviations were calculated for numeric/quantitative variables. Chi square test was carried out to determine relationships between categorical variables and statistical significance was said to be present at *P* value of less than 0.05. The independent variables were socio-demographic characteristics of women (age category, level of education, partner’s/husband’s education, number of living children, and religion). The dependent variables were uptake of any family planning method and pattern of use of family planning methods.

Qualitative data analysis commenced with verbatim transcription of audio recordings and review of transcripts with hand-written notes to ensure completeness of information and inclusion of non-verbal responses. Pre-conceived themes were then generated from the interview guide to develop a coding framework. The four transcripts were then read to achieve familiarization and identify any themes that were not in the coding framework. The final coding framework was then applied to all 4 transcripts. The themes in the final coding framework are: i) Perceptions of family planning and artificial methods of contraception, ii) Attitude towards artificial methods of family planning and methods commonly used, iii) Partner involvement in family planning.

## Result

**Fig. 1:**
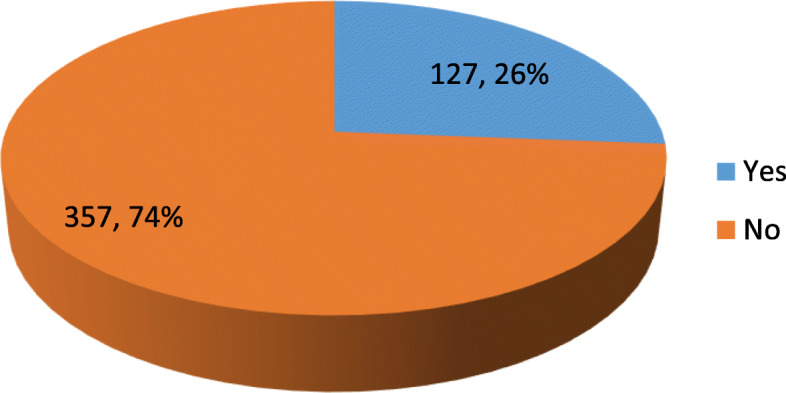
Proportion of respondents using a method of family planning

Table 1 shows the socio-demographic characteristics of the respondents. Mean age of the respondents was 29.2 ± 6.1. Over half (57.4%) of the respondents were of the Roman Catholic denomination. Majority of the respondents and their spouses had at least secondary education. Greater proportion (71.9%) of the respondents had less than five children. Almost all the respondents (91.5%) were in a monogamous family setting. The husband/partner was the household head and decision maker on health matters for most of the respondents (94.6 and 92.8% respectively). Figure 1 shows that only 26.2% of the respondents were using a method of family planning. In Table 2, among those using a method of family planning, majority were using the natural method and amongst the 43% who reported using artificial methods, 32.7% used condoms, 27.3% used implant while 23.64 and 16.4% used injectables and pills respectively. Most of those using any method of family planning did so consistently (79.53%) and had used a method for at least 6 months (77.17%). Older age (AOR = 1.7, 95%CI = 1.01–3.00), having more than five children (AOR = 1.7, 95%CI = 1.05–2.83), and attaining secondary education by husband (AOR = 2.0, 95%CI = 1.05–3.92), and respondent (AOR = 3.3, CI = 1.60–6.96) were predictors of use of family planning methods (Table 3).

**Table 1:** Socio-demographic and household characteristics of respondents

Variable	***n*** = 484	(%)
**Age (Mean ± SD)**	29.2 ± 6.1	
**Religious denomination**		
Roman Catholic	278	57.4
Other denominations	206	42.6
**Educational level**		
Primary and less	99	20.5
Secondary and more	385	79.5
**Spouse’s Educational level**		
Primary and less	104	21.5
Secondary and more	380	78.5
**Number of living children**		
< 5 children	348	71.9
≥ 5 children	136	28.1
**Type of household**		
Monogamous	443	91.5
Polygamous	41	8.5
**Household head**		
Husband/partner	458	94.6
Others*	26	5.4
**Decision maker on health matters**		
Husband/partner	449	92.80
Others*	35	7.20

*father in-law, mother in-law, Self, Parents

**Table 2:** Pattern of use of family planning methods

Variable	***n*** = 127	%
**Method used**		
Natural method	72	56.60
Artificial method	55	43.40
**Type of artificial method used**		
Pills	9	16.36
Barrier	18	32.72
Injectable	13	23.64
Implant	15	27.27
**Frequency of use of any family planning method**		
Consistent	101	79.53
When affordable	3	2.36
If I remember	11	8.66
If partner permits	12	9.45
**Duration of use of any method of family planning**		
< 6 months	29	22.83
6- < 12 months	17	13.39
1–5 years	61	48.03
> 5 years	20	15.75

**Table 3:** Predictors of uptake of any family planning method

Independent variable	AOR	CI
**Age in years**		
≥ 35 years	1.7	1.01–3.00
< 35 years	1	
**Respondent’s education**		
≥ Secondary	3.3	1.60–6.96
≤ primary	1	
**Spouse’s education**		
≥ Secondary	2.0	1.05–3.92
≤ primary	1	
**Number of living children**		
≥ 5 children	1.7	1.05–2.83
< 5 children	1	
**Religion**		
Other denominations	1.1	0.73–1.70
Roman catholic denomination	1	

### FGD result

#### Perceptions of family planning and artificial methods of contraception

Majority of the respondents demonstrated a good knowledge of family planning and its benefits. They reported that family planning is used for child spacing and is beneficial for child health and family size. Some supporting quotes are: “*Family planning is a way of delaying pregnancy so that the child grows before another comes*” (Female).

“*It* (family planning) *helps women take care of the children and also helps them decide the number of children to have*” (Female).

However, contraception is not practiced by families in the community due to fatalistic reasons and misconceptions about side effects of modern methods of contraception. One respondent said:

“*Some families in this community do not accept family planning because they believe it causes bleeding and barrenness*” (Female).

Some respondents stated that people may not practice family planning because they believe that limiting family size has negative implications for economic and food productivity of the household. Also, family planning goes against God’s plan of fruitfulness and the church does not support modern methods of contraception.

“*Villagers say it is not good, people should deliver all the children in their womb, be it 10 or 20. Children should fill the home so that farming would be more productive and there will be food everywhere. If family planning is done, there will be no productivity and thus hunger”*(Female).

The respondents also reported the position of their religious denominations about family planning. Some affirmed that the church supports only the natural method of family planning. “*Church says if you stop it will make church members to be small. The bible says go and fill the earth so give birth to all in your womb. Church says go natural but modern methods are not natural*” (Female).

The men also appeared to share similar views as the women with respect to family planning and contraception. For instance, they perceived limiting family size as interfering with God’s plan for procreation. They were also of the opinion that having more children is a form of assurance in the event of death. In the words of one of the men,

“*Family planning is not good. If God has said you’ll have many children and you do family planning and have only two, if one dies, you’ll be left with only one. We should allow God so that we can have the number of children he wants us to have”*( Male)*.*

Participants also highlighted that women who practiced family planning were perceived as being selfish and denying men their rights and reported as follows:

“*The men do not accept it, they feel they’re being denied their right”* (Female).

Others stated that it is believed that women who make use of family planning do not have feelings for their spouses. One of the respondents quoted as follows: *“Some people do not accept family planning. They believe that women who have done family planning do not have feelings for their husbands”* (Male)*.*

### Attitude towards artificial methods of family planning and methods commonly used

Attitude towards artificial methods of family planning was reported to be generally poor. The attitudes of the respondents towards family planning arise from perceptions about side effects which appeared to vary depending on the type of contraceptive. One of the male respondents commented as follows:

“*Men feel negative impact, for example, Condom they say it can burst and if it bursts can lead to disease; if injectable is used it leads to difficulty in getting pregnant; and IUD leads to bleeding”* (Male)*.*

However, the women had genuine concerns about the side effects of modern methods of contraception that influence their attitude. One of the women said:

*“It is good but has much side effects, fat, ceases menses and if it comes there’s heavy flow, enlargement of breast”* (Female)

The respondents also revealed the fact that community support for family planning is lacking. Some men in the community could go to the extent of sending their wives away because of family planning. One of the supporting quotes is shown below:

“*Villagers say husbands won’t agree when wife does it, and when some wives do it their husbands chase them away”* (Male)*.*

Although a few of the participants stated that they use artificial methods of family planning, majority of them preferred and used natural methods. Some of the reasons for the preference of natural method were perception of safety, effectiveness and absence of side effects. Additionally, some women stated that their partners did not oppose natural methods.

“ … *Natural method is safer and has no side effect”*(Male)

“ … *Natural method is what I will use, it is the best”*(Male)

Women who used modern methods did so for various reasons. Some had no ugly experiences or adverse effects from the methods they used and therefore continued using these methods. Some of their quotes are shown below:

*“ … I use Condom, it has no side effects and my husband does not complain”*(Female);

*“ … I use implants and no much side effects, once removed, I take in”* (Female)

### Partner involvement in family planning

The respondents reported that family planning is not discussed except among literate men. Most of the men do not want to discuss about family planning especially when they have not completed their family size. Many men do not support nor give approval for their wives to practice family planning. Some supporting quotes are:

“*I have a friend that has six children but as soon as she mentioned family planning to her husband, he shouted at her and bluntly refused”*. (Female)

“*The men see having as many children as they want as their right and so any one talking about family planning is like an enemy”*(Male)

Although discussions around family planning in the home are raised by women, a few of the participants affirmed that when they discuss family planning with their husbands they are supportive of their choices, even if it takes a while to happen. “*My husband and I talk about it and decide on which method to use …*. *Females are always the ones that bring it up”*(Female)

*“My husband told me it is good to wait for 2-3 years so he will save enough and I agreed. My husband pays for it”*.(Female)

## Discussion

This study assessed the perception, pattern of use, and partner support for family planning and equally identified predictors of uptake of family planning.

This study revealed that only few of the respondents were using any method of family planning at the time of the survey and majority of these were using the natural method. Previous studies have also reported low family planning use [8–16]. Uptake of family planning is still low in developing countries including Nigeria [5]. The low uptake reported in the quantitative survey was also affirmed by the FGD participants. Although all of the women who participated in the FGDs perceived family planning as beneficial, they reported that most families did not practice it. FGD respondents expressed various reasons for not using contraceptives. Some of the reasons given for non-use of family planning methods include: desire for more children, fear of side effects, religious beliefs and the fact that reduction in family size has a negative effect on economic and food productivity. This shows the need to identify and intensify interventions which are multi-sectoral to increase uptake of family planning.

Use of natural method by majority of the respondents is a pointer to the fact that these women are probably having fears about side effects of modern methods. The FGD respondents also concurred with the fact that natural method of family planning is mostly encouraged in the community with majority having a generally poor attitude towards the artificial methods. Consequently, it is necessary that information on various methods of family planning including their mechanisms of action be included in the content of the health education usually delivered to women both during antenatal, post natal clinics, and during community outreaches. It may also be of benefit to pay attention to teaching women about the natural family planning method so that those who choose this method can use it correctly and effectively. Additionally, unavailability and cost of artificial methods could also play a role in the preference for natural methods. Among those using a method of family planning, majority were consistent in using a method. This finding disagrees with a study carried out in Rivers State which showed that only 22.6% of respondents used a method consistently [17]. This disparity may be due to the fact that the study in Rivers State assessed only use of modern methods of contraceptives. It’s been shown that women’s use-related behaviors, especially the consistency with which they take pills is associated with effectiveness of contraceptives [18].

The independent variables found to predict the use of family planning methods in this study were age, respondents’ education, husband’s education, and number of living children. Those that were ≥ 35 years were 1.7 times more likely to use a method of family planning than those who were less than 35 years. This may be because older women might have completed their family size considering the fact that women marry early in the African culture, particularly those who live in the rural areas [5, 19, 20].

Husband’s education was positively correlated with current use of family planning for those with at least secondary-level of education compared to those with a primary-level of education or less. This is similar to the finding in a study carried out in Pakistan which reported that husband’s education positively correlated with current contraceptive use among women [21]. The FGD further supports the fact that education has an influence on family planning use as some women reported that family planning discussions come up mainly among literate men. This study further revealed that men were the main decision makers on health matters. This was further buttressed by the FGD respondents. Therefore, the importance of men’s education cannot be overemphasized because it has a huge role to play in improving uptake of family planning and other health services. Partner support for family planning including spousal communication is a crucial factor that can influence use [15, 21, 22]. Evidence shows that men’s awareness of, and support for, use of contraceptives are associated with their spouses’ desire to use contraception [23]. It is therefore essential to address the problem of literacy among men while also emphasizing female education. Some FGD respondents however stated that they get their husband’s support even if it takes some time to convince them. This shows the need for persistence in the efforts to improve male involvement in family planning.

Those that had five or more children were more likely to be family planning users. This finding was also supported by the FGD respondents who believed that women should have as many children as they want, it is therefore unlikely for women who have few children or who have not achieved their desired family size to use a method of family planning. A Ghanaian study found a positive predictive correlation between number of living children and uptake in a study on correlates of contraceptive use among women of reproductive age [24].

Although religion has been reported as a factor that affects uptake of family planning [9, 12, 25] and our FGD also reported religion as a reason for non-use, paradoxically, religion was not found to be a predictor of family planning use in this study. This could be because individuals are beginning to take decisions that are beneficial to their health without being clouded by the decision of third parties.

One strength of this study is that it employed a mixed method design which makes it possible to understand better the topic under study. This study was however carried out in rural communities and therefore limits the extent to which the findings can be generalized.

## Conclusion

The study has shown that natural family planning methods were most used with age, parity, self and partner education as predictors of current use of any method. Religion on the contrary was not found to be a predictor of family planning. Desire for more children, fear of side effects, religious beliefs and the perceived negative effect of small family size on economic and food productivity are some reasons why uptake of family planning is low in rural communities. Improving uptake of family planning methods in these rural communities will require proper demographic targeting as well as debunking fatalistic views, and cultural and religious myths around family planning. Family planning interventions should therefore target older and grand-multiparous women. It is also necessary to explore ways of improving the educational status of men and women in the community.

## Data Availability

The datasets used and/or analysed during the current study are available from the corresponding author on reasonable request.

## Abbreviations

SPSS: Statistical Package for Social Sciences (IBM-SPSS)
IUCD: Intrauterine Contraceptive Device
LGA: Local Government Area
FGD: Focus Group Discussion

## References

[1] World Health Organisation. Family planning / contraception factsheet. http://www.who.int/mediacentre/factsheets/fs351/en/ Accessed July 2016.

[2] WHO | The ABC’s of family planning. WHO. World Health Organization; 2013.

[3] Pack AP, McCarraher DR, Chen M, Okigbo CC, Albert LM, Wambugu S (2014). Factors associated with unmet need for modern contraception in post-conflict Liberia. Afr J Reprod Health.

[4] United Nations, Department of Economic and Social Affairs. Contraceptive Use by Method 2019 Data Booklet. 2019. www.un.org › sites › files › files › documents › Jan › u...Accessed March 5 2020.

[5] National Population Commission. Nigeria Demographic And Health Survey 2018. 2018.

[6] Okunade K, Daramola E, Ajepe A, Sekumade A (2016). A 3-year review of the pattern of contraceptive use among women attending the family planning clinic of a university teaching hospital in Lagos, Nigeria African. J Med Heal Sci.

[7] Akamike IC, Mbachu C, Onwasigwe C, Okedo-Alex I, Eze I, Eze N. Role of community resource persons in improving use of modern family planning methods among women of reproductive age in a rural area in Ebonyi state, Nigeria. Int J Health Plann Manage. 2019:1–10. 10.1002/hpm.2746 30734967

[8] Nwachukwu I, Obasi OO (2008). Use of modern birth control methods among rural communities in Imo state, Nigeria. Afr J Reprod Health.

[9] Oye-Adeniran BA, Adewole IF, Umoh A, Oladokun A, Gbadegesin A, Ekanem EE (2006). Community-based study of contraceptive behaviour in Nigeria. Afr J Reprod Health.

[10] Sanusi A, Akinyemi O, Onoviran O (2015). Do knowledge and cultural perceptions of modern female contraceptives predict male involvement in Ayete, Nigeria?. Afr J Reprod Health Women’s Health Act Res Center.

[11] Avidime S, Aku-Akai L, Mohammed A, Adaji S, Shittu O, Ejembi C (2014). Fertility intentions, contraceptive awareness and contraceptive use among women in three communities in northern Nigeria. Afr J Reprod Health Women’s Health Act Res Center.

[12] Egede JO, Onoh RC, Umeora OUJ, Iyoke CA, Dimejesi IBO, Lawani LO (2015). Contraceptive prevalence and preference in a cohort of south-east Nigerian women. Patient Prefer Adherence.

[13] Doctor H, Findley SE, Afenyadu GY, Uzondu C, Ashir GM (2013). Awareness, use, and unmet need for family planning in rural northern Nigeria. Afr J Reprod Health.

[14] Wendo BM. Barriers to uptake of long term and permanent family planning methods among HIV infected postpartum mothers in Kenyatta national hospital. Department of Obstetrics and Gynaecology, University of Nairobi; 2013. http://erepository.uonbi.ac.ke/handle/11295/71463?show=full. Accessed Jan 2017.

[15] Choge MC. Contraceptive uptake among women of reproductive age in Kakuma refugee camp in Turkana county, Kenya. Kenyatta University; 2012. http://ir-library.ku.ac.ke/handle/123456789/7680. Accessed April 2020.

[16] Namazzi G. Missed Opportunities for Modern Family Planning Services among women attending Child health clinics in Iganga/ Mayuge Demographic Surveillance site. 2013. www.musphcdc.ac.ug › files › pdf ›. Accessed April 2020.

[17] Osaro BO, Tobin-West CI, Mezie-Okoye MM (2017). Knowledge of modern contraceptives and their use among rural women of childbearing age in Rivers state Nigeria. Ann Trop Med Public Heal.

[18] Benagiano G (1992). Introduction: enhancing oral contraceptive compliance and efficacy. Adv Contracept.

[19] National Bureau of Statistics and UNICEF. Multiple Indicator Cluster Survey 2016–17, Survey Findings Report. 2017.

[20] UNFPA & UNICEF. Child Marriage in West and Central Africa: At a Glance. September 2018.

[21] Azmat SK, Ali M, Ishaque M, Mustafa G, Hameed W, Khan OF (2015). Assessing predictors of contraceptive use and demand for family planning services in underserved areas of Punjab province in Pakistan: results of a cross-sectional baseline survey. Reprod Health.

[22] Sileo KM (2015). Determinants of family planning service uptake and use of contraceptives among postpartum women in rural Uganda. Int J Public Health.

[23] Ezeanolue EE, Iwelunmor J, Asaolu I, Obiefune MC, Ezeanolue CO, Osuji A (2015). Impact of male partner’s awareness and support for contraceptives on female intent to use contraceptives in Southeast Nigeria. BMC Public Health.

[24] Nketiah-Amponsah E, Arthur E, Abuosi A (2012). Correlates of contraceptive use among Ghanaian women of reproductive age (15-49 yrs). Afr J Reprod Health.

[25] Ankomah A, Anyanti J, Adebayo S, Giwa A (2013). Barriers to contraceptive use among married young adults in Nigeria : a qualitative study. Int J Trop Dis Health.

